# Role of Oxidative Stress in the Suppression of Immune Responses in Peripheral Blood Mononuclear Cells Exposed to Combustible Tobacco Product Preparation

**DOI:** 10.1007/s10753-017-0602-9

**Published:** 2017-06-02

**Authors:** Subhashini Arimilli, Eckhardt Schmidt, Brad E. Damratoski, G. L. Prasad

**Affiliations:** 1 Department of Microbiology & Immunology, Wake Forest University Health Sciences, Room 2N-052, 575 Patterson Avenue, Winston-Salem, NC 27101 USA; 2 RAI Services Company, Winston-Salem, NC USA; 3 Department of Regenerative Medicine, Wake Forest University Health Sciences, Winston-Salem, NC USA

**Keywords:** tobacco product preparations, *N*-acetylcysteine, PBMCs, LPS, cytotoxicity, cytokines, cytolysis

## Abstract

Cigarette smoking is a major risk factor for several human diseases. Chronic inflammation, resulting from increased oxidative stress, has been suggested as a mechanism that contributes to the increased susceptibility of smokers to cancer and microbial infections. We have previously shown that whole-smoke conditioned medium (WS-CM) and total particulate matter (TPM) prepared from Kentucky 3R4F reference cigarettes [collectively called as combustible tobacco product preparations (TPPs)] potently suppressed agonist-stimulated cytokine secretion and target cell killing in peripheral blood mononuclear cells (PBMCs). Here we have investigated the role of oxidative stress from TPPs, which alters inflammatory responses *in vitro*. Particularly, we investigated the mechanisms of WS-CM-induced suppression of select cytokine secretions in Toll-like receptor (TLR) agonist-stimulated cells and target cell killing by effector cells in PBMCs. Pretreatment with *N*-acetyl cysteine (NAC), a precursor of reduced glutathione and an established anti-oxidant, protected against DNA damage and cytotoxicity caused by exposure to WS-CM. Similarly, secretion of tumor necrosis factor (TNF), interleukin (IL)-6, and IL-8 in response to TLR-4 stimulation was restored by pretreatment with NAC. Target cell killing, a functional measure of cytolytic cells in PBMCs, is suppressed by WS-CM. Pretreatment with NAC restored the target cell killing in WS-CM treated PBMCs. This was accompanied by higher perforin levels in the effector cell populations. Collectively, these data suggest that reducing oxidative stress caused by cigarette smoke components restores select immune responses in this *ex vivo* model.

## INTRODUCTION

Cigarette smoking is a major risk factor for serious diseases such as cancer, chronic obstructive pulmonary disease (COPD), and cardiovascular diseases [1]. Cigarette smoke is a free radical-rich aerosol that contains thousands of chemicals, which can be partitioned into gas-vapor phase and particulate phases [2]. One of the mechanisms through which cigarette smoke exerts its adverse effects is through reactive oxygen species (ROS) present in smoke, which cause oxidative stress and induce local and systemic inflammatory responses [3]. Previous studies have suggested that exposure to cigarette smoke leads to an increased susceptibility to infections by influenza virus and *Mycobacterium tuberculosis* and significant changes in cellular immune responses [4, 5].

Given the complexity of cigarette smoke aerosol, several different preparations/fractions of cigarette smoke were utilized in different *in vitro* models to elucidate the mechanism of the adverse pathophysiological effects due to smoking [6]. For example, freshly generated smoke is bubbled through aqueous buffers/cell culture media to generate cigarette smoke extracts (CSE), which we have termed whole-smoke conditioned media (WS-CM); these preparations primarily contain water-soluble carbonyls, including acrolein, acetaldehyde, formaldehyde, 1,3 butadiene, ammonia, and nicotine. The particulate phase of cigarette smoke is collected on Cambridge filter pads and dissolved in dimethyl sulfoxide (DMSO), and is termed total particulate matter (TPM). Nicotine, tobacco-specific nitrosamines, organic amines, and other toxicants make up TPM. Direct treatment of cells with either TPM or WS-CM offers a simpler and yet useful approach than complex approaches that employ direct exposure of cells using air-liquid interphase systems, and have been extensively utilized [7]. We have collectively referred to WS-CM, TPM, and other preparations from reference tobacco products (such as 3R4F and 2S3 moist snuff) as tobacco product preparations (TPPs) [8].

Previously we and others showed in cell culture systems that the combustible TPPs including WS-CM and TPM were more cytotoxic and genotoxic than non-combustible TPPs [8–10]. Lipopolysaccharide (LPS), an endotoxin in the membranes of gram-negative bacteria, binds to Toll-like receptor (TLR)-4 of the cell and effectively activates immune responses by inducing expression of proinflammatory cytokines [11]. TLR-stimulated inflammatory responses such as cytokine expression and secretion were significantly reduced upon treatment with the aqueous extract of cigarette smoke (CSE and WS-CM) [12–14].

Constituents of combustible TPPs have been shown to be potent pro-oxidants, which perturb the balance of intracellular pro- and anti-oxidants and consequently impact several biological pathways including inflammation [15]. Oxidants/reactive oxygen species (ROS) found in cigarette smoke are major contributors in mediating an inflammatory state in the pathogenesis of diseases such as COPD and lung cancer [16]. *In vitro* studies with primary T cells exposed to CSE which is a ROS source have revealed the induction of T cell apoptosis and decreased T cell proliferation [17].

Previous studies have shown that *N*-acetylcysteine (NAC) produced immunomodulatory effects through its actions as a scavenger of superoxide radicals and ROS [18–22]. Deacetylation of NAC yields l-cysteine, which is a precursor to glutathione synthesis. Glutathione is an intracellular anti-oxidant, and plays a major role in protecting cells against oxidative stress and DNA damage [18, 23]. Treatment with NAC was shown to reverse cigarette smoke-induced myocardial infarction by inhibiting inflammation and oxidative stress in a rat model [24] and prevented lung tumors in mice [25]. Furthermore, NAC possesses anti-inflammatory properties [26] and has been suggested as a potential treatment option for cancer and prevent genetic mutations from occurring *via* reducing the role of reactive oxidative species in inducing mutations [23].

We have previously shown that TPM and WS-CM cause a dose-dependent increase in DNA damage and cell death and a dose-dependent reduction in secreted cytokines, intracellular cytokines, and cytolysis in PBMCs [8, 12]. Here we investigated the role of oxidative stress caused by combustible TPPs in suppression of immune functions and how one can restore select immunosuppression by reducing oxidative stress in an *ex vivo* model.

## MATERIALS AND METHODS

### TPPs and Cytotoxicity Measurement

WS-CM and TPM were prepared from 3R4F cigarettes as described previously [8]. Partial characterization of WS-CM was performed at Labstat International, Kitchener, Canada, and it included analyses of several known toxicants of cigarette smoke (Table 1). Cytotoxicity was measured after exposing PBMCs to various concentrations of WS-CM or TPM for 3 or 24 h and staining the exposed cells with 7-aminoactinomycin D (7AAD), which labels dead cells. 7AAD-positive cells were measured by flow cytometry and analyzed by the FlowJo software (Tree Star, Ashland, OR) at various doses based on the equi-nicotine unit paradigm. The EC_50_ value of WS-CM and TPM was defined as the concentration at which 50% of the cells were no longer viable in a 24-h 7AAD assay. The EC_50_ value of WS-CM and TPM was determined to be 1.56 and 2.58 μg/mL of equi-nicotine units, respectively [8]. To measure the effect of NAC on the cytotoxicity, PBMCs were pre-incubated with and without 5 mM NAC for 1 h and subsequently exposed to different concentrations of WS-CM plus 5 mM NAC for 24 h. PBMCs were washed and stained with 7AAD and measured by flow cytometry.

**Table 1:** Chemical Analysis of WS-CM

Tobacco constituents of WS-CM	Units	Mean	SD
Nicotine	(μg/mL)	9.2	0.8
Nornicotine	(μg/mL)	BDL	–
Anabasine	(μg/mL)	BDL	–
Myosmine	(μg/mL)	BDL	–
Anatabine	(μg/mL)	BDL	–
Un-ionized nicotine	(μg/mL)	4.48	0.7
Ammonia	(μg/mL)	9.19	0.6
Nitrosonornicotine (NNN)	(pg/mL)	1716	717
Nitrosoanatabine (NAT)	(pg/mL)	1506	1145
Nitrosoanabasine (NAB)	(pg/mL)	306	234
4-(*N*-nitrosomethylamino)-1-(3-pyridyl)-1-butanone (NNK)	(pg/mL)	2470	2013
NDMA	(ng/mL)	2.45	–
NPIP	(ng/mL)	BDL	–
NPYR	(ng/mL)	BDL	–
NMOR	(ng/mL)	BDL	–
Acrylamide	(ng/mL)	28.5	2
Nitrite	(μg/mL)	BDL	–
Ethyl carbamate	(ng/mL)	BDL	–
*N*-nitrosodiethanolamine (NDELA)	(ng/mL)	NQ	–
*N*-nitrosodiisopropanolamine (NDiPLA)	(ng/mL)	BDL	–
*N*-nitrososarcosine	(ng/mL)	BDL	–
Coumarin	(ng/mL)	2.02	0.58
Formaldehyde	(μg/mL)	0.621	0.07
Acetaldehyde	(μg/mL)	73.6	1.27
Acetone	(μg/mL)	34.9	0.62
Acrolein	(μg/mL)	0.152	0.01
Propionaldehyde	(μg/mL)	4.28	0.06
Crotonaldehyde	(μg/mL)	1.28	0.05
Methyl ethyl ketone	(μg/mL)	10	0.19
Butyraldehyde	(μg/mL)	0.475	0.02
pH	(unit)	7.99	0.06
Osmolarity	(mOsm/L)	280	0.58

### PBMC Isolation and Stimulation

PBMCs were isolated from fresh blood as described previously [27]. Isolated PBMCs were cryopreserved for further use. Cryopreserved PBMCs were thawed and then pre-incubated with and without 5 mM NAC for 1 h before exposure to different concentrations (equi-nicotine units) of WS-CM or TPM, and with and without 5 mM NAC, for 3 h in RPMI 1640 media containing 10% fetal bovine serum with 1% l-glutamine, 1% penicillin, and streptomycin (RPMI complete medium). Cells were then washed and stimulated with 10 μg/mL LPS for 24 h. After the 24-h stimulation, the cell culture supernatants were collected for analysis of secreted cytokines.

### Cytometric Bead Array Assay

PBMC culture supernatants were analyzed using a Cytometric Bead Array (CBA) Human Inflammation kit or Th1/Th2 Human Cytokine kit (BD Biosciences, San Jose, CA) to measure interferon (IFN)-γ; tumor necrosis factor (TNF); and interleukin (IL)-1β, IL-6, IL-8, and IL-10 by flow cytometry according to the manufacturer’s instructions.

### ELISA Assay

Whenever the Th1/Th2 Human Cytokine kit was used, IL-8 was measured by enzyme-linked immunosorbent assay (ELISA) by using a kit from R&D Systems (Minneapolis, MN) according to the manufacturer’s protocol.

### Intracellular H2AX and Perforin Measurement

PBMCs were pre-incubated with and without 5 mM NAC for 1 h and subsequently exposed to different concentrations of WS-CM plus 5 mM NAC for 24 h. PBMCs were washed, fixed, and permeabilized with BD Biosciences Cytofix/Cytoperm buffer for 20 min on ice in the dark. Cells were then stained with ser-139 phosphorylated H2AX (γ-H2AX-Alexa Fluor® 647, BD Biosciences) to measure DNA double-stranded breaks. To measure perforin levels, PBMCs were pre-incubated with and without 5 mM NAC for 1 h and subsequently exposed to different concentrations of WS-CM plus 5 mM NAC for 3 h. PBMCs were washed, fixed, permeabilized, and stained with anti-perforin-fluorescein isothiocyanate monoclonal antibody for an additional 30 min on ice. Perforin-positive cells were measured by flow cytometric analysis, and the data were analyzed using the FlowJo software.

### Oxidative Stress Assay

To measure oxidative stress, PBMCs were pre-incubated with and without 5 mM NAC for 20 h and subsequently exposed to different concentrations of WS-CM or TPM along with 5 mM NAC for 1 h and labeled with the CellROX® Deep Red Reagent kit (Life Technologies, Grand Island, NY) according to the manufacturer’s instructions. Pre-incubation of PBMCs with 5 mM NAC for 1 h showed no effect on oxidative stress. CellROX reagent preferentially binds to superoxide and hydroxyl radicals, and is essentially non-fluorescent in a reduced state but exhibits a strong fluorogenic signal upon oxidation, thereby providing a reliable measure of ROS in live cells. The fluorescence was measured by flow cytometry, and the data were analyzed using the FlowJo software.

### Target Cell Killing Assay

K562 target cells were purchased from ATCC (Manassas, VA) and grown in RPMI complete medium. K562 cells were labeled with carboxyfluorescein succinimidyl ester (CFSE). PBMCs were pre-incubated with and without 5 mM NAC and exposed to different concentrations of WS-CM for 3 h; cells were washed and mixed with CFSE-labeled K562 cells at a density of 100,000 cells/well (target/effector ratio 1:15). Cell co-cultures were incubated at 37 °C for an additional 5 h. Immediately after the incubation period, cells were stained with 7AAD and the killing of CFSE-labeled K562 target cells was evaluated by flow cytometry and the data were analyzed using the FlowJo software.

### Statistical Comparisons

Results are presented as the mean ± the standard error of the mean (four donor samples). Treated and control samples were compared using *t* tests. The statistical significance was indicated by *P < 0.05, **P < 0.005, and ***P < 0.0005.

## RESULTS

Partial characterization of WS-CM was performed, and the mean and standard deviations of WS-CM tobacco constituents from three different batches were calculated and are presented in Table 1. The mean value of nicotine was 9.2 μg/mL, and from this value, the equi-nicotine units were calculated when different doses of WS-CM were used in the experiments. Tobacco-specific nitrosamines, nitrosonornicotine (NNN), nitrosoanatabine (NAT), and 4-(*N*-nitrosomethylamino)-1-(3-pyridyl)-1-butanone (NNK) were detected in WS-CM. Of these three compounds, NNK was detected at a higher level at 2470 pg/mL, followed by NNN at 1716 pg/mL and NAT at 1506 pg/mL. Carbonyl compounds known to be biologically reactive and contribute to ROS generation, such as aldehyde and ketone levels, are also measured in this panel, and their levels are indicated in Table 1. Several other constituents tested in this panel were shown to be below the detection limit (BDL).

**Fig. 1:**
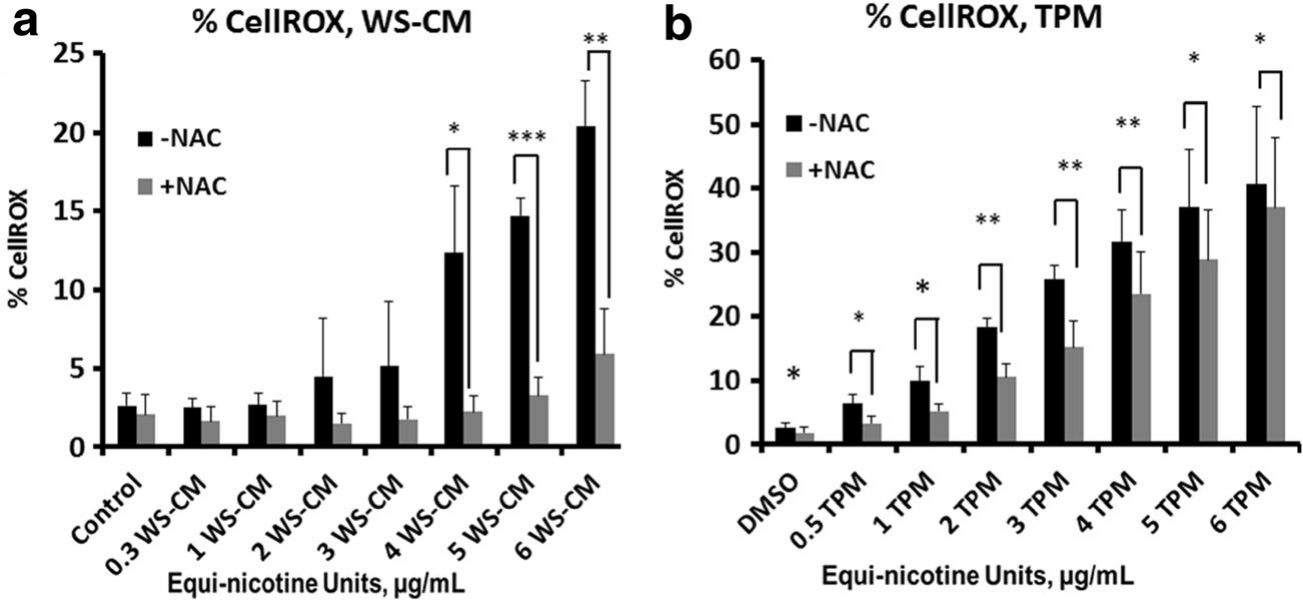
Effects of NAC on oxidative stress in WS-CM- and TPM-exposed PBMCs. PBMCs were pretreated with or without NAC and followed by co-incubation with different concentrations of WS-CM (**a**) or TPM (**b**). Oxidative stress was measured using a CellROX® kit and flow cytometry. Each point represents the mean ± SD *error bars* of four donors from a representative experiment.

To determine the degree of burden of oxidative stress induced by WS-CM or TPM, we used a CellROX assay that directly measures ROS in live cells. PBMCs were pretreated with NAC (or without) and exposed to increasing amounts of WS-CM or TPM. A dose-dependent increase in percent CellROX was observed with both WS-CM and TPM. ROS levels, as measured by CellROX, were greatly reduced after the addition of NAC in a dose-dependent manner, particularly at concentrations over 2 μg/mL WS-CM and TPM (expressed as equi-nicotine units) concentrations (Fig. 1). At a 5 μg/mL equi-nicotine unit of WS-CM, the amount of ROS detected without NAC was 44%, but was lowered to 13% (statistically significant difference) in the presence of NAC (Fig. 1a). With TPM concentrations ranging from 0.5 to 4 μg/mL, the amount of ROS in PBMCs that was decreased by addition of NAC was also statistically significant (Fig. 1b). With the TPM samples, there were statistically significant differences with the addition of NAC at the lower concentrations of TPM as compared with WS-CM at similar, equi-nicotine unit concentrations.

**Fig. 2:**
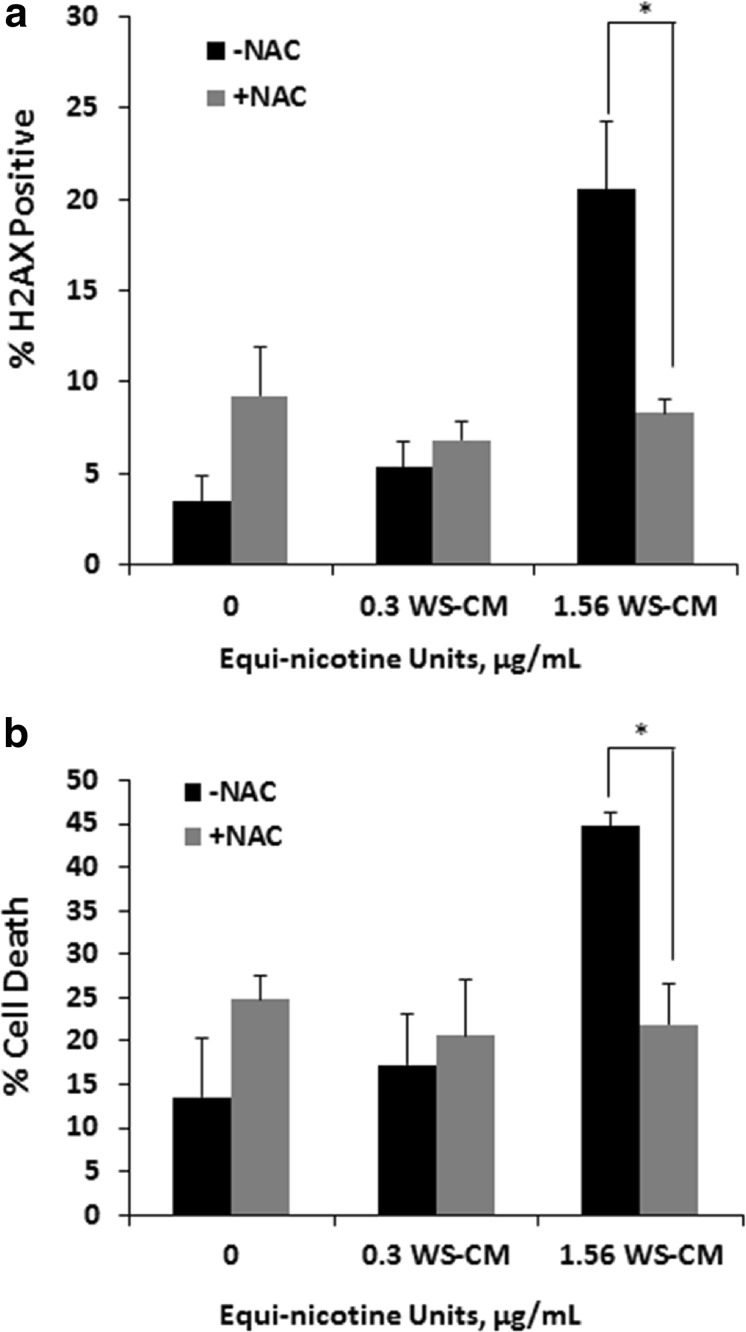
Recovery of cytotoxic effects of WS-CM-exposed PBMCs pretreated with NAC. PBMCs were pretreated with or without NAC for 1 h followed by co-incubation with WS-CM for 24 h. DNA damage was measured by H2AX staining using flow cytometry (**a**) and cell death was measured by 7AAD staining (**b**). Each point represents the mean ± SD *error bars*. Data were derived from samples provided by four different donors; shown here are results from a representative experiment. *P < 0.05.

In our previous studies, we used different concentrations of WS-CM exposed to PBMCs at 3 h and showed that there is no significant cytotoxicity [28]. Hence we selected two concentrations of WS-CM to measure cytotoxicity and other endpoints in the current study and compared these results to the control. We assessed whether NAC-mediated reduction of ROS inhibits DNA damage and death of PBMCs caused by exposure to WS-CM. PBMCs were pretreated with or without NAC followed by co-incubation with WS-CM. We measured DNA damage by H2AX labeling and cell death by 7AAD labeling. The percent of H2AX-positive cells increased in a dose-dependent fashion with WS-CM treatment [8]. At 1.56 μg/mL WS-CM exposure, 20% cells were positive for H2AX; however, with the treatment of NAC, the percent of H2AX-positive cells was reduced to 8% (Fig. 2a). Similarly, the addition of NAC significantly decreased the number of dead PBMCs from 45 to 23% at 1.56 μg/mL WS-CM exposure (Fig. 2b).

**Fig. 3:**
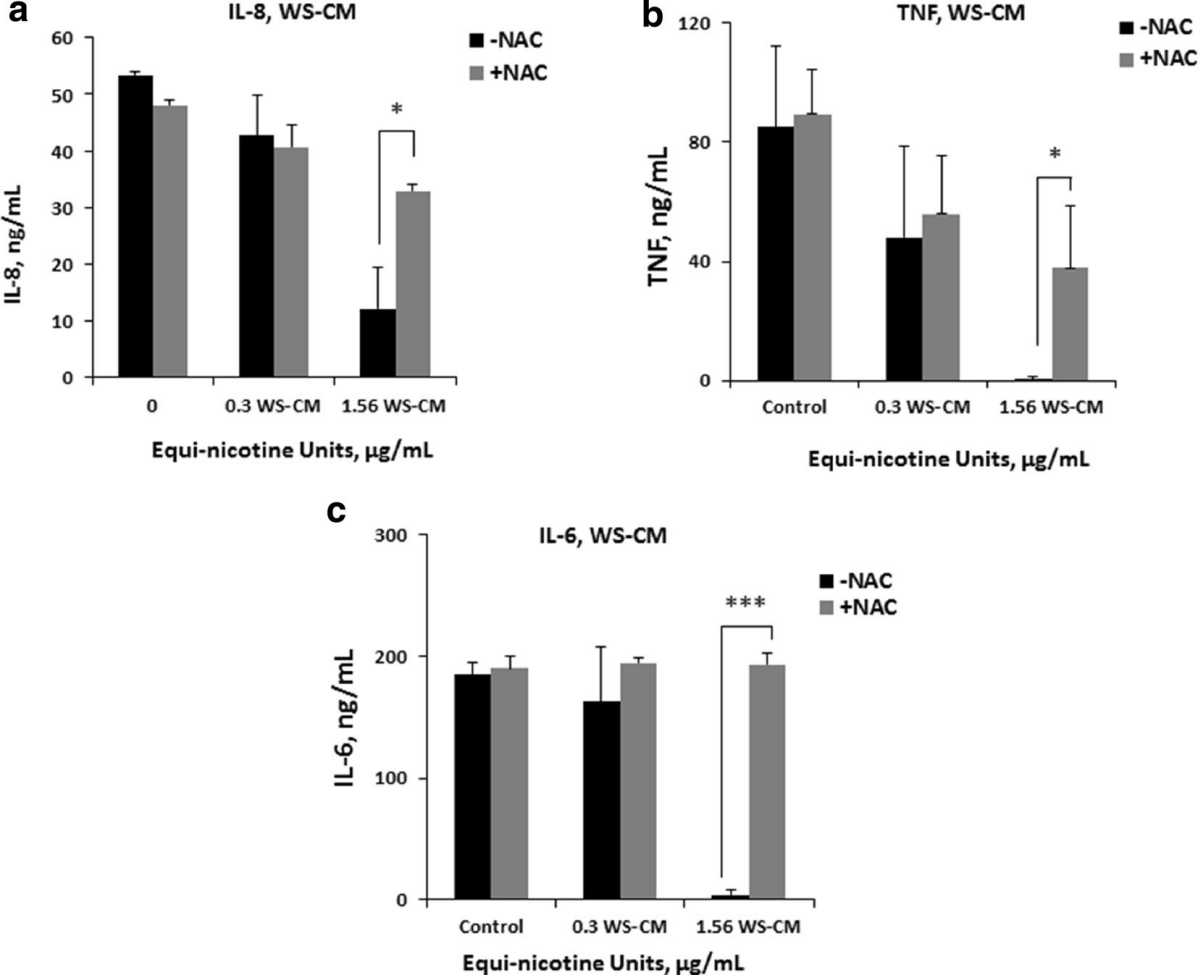
Recovery in cytokine secretion of PBMCs with NAC after WS-CM exposure. PBMCs were treated with or without NAC for 1 h followed by co-incubation with WS-CM for 3 h and LPS stimulation for 24 h. Levels of IL-8 (**a**), TNF (**b**), and IL-6 (**c**) cytokines in the cell culture supernatants were obtained from PBMCs. Each point represents the mean ± SD *error bars* of four donors from a representative experiment. Statistical significance is indicated by *P < 0.05; ***P < 0.0005.

**Fig. 4:**
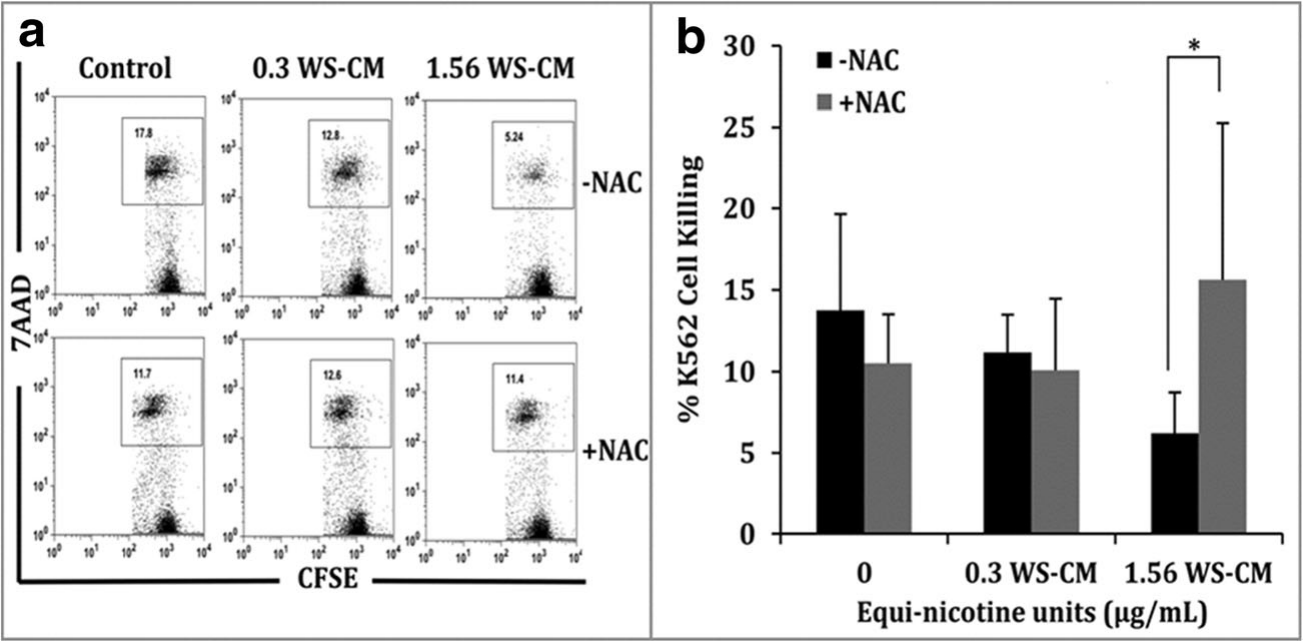
Recovery in cytolysis of effector cell PBMCs with NAC after WS-CM treatment. PBMCs were exposed to indicate concentrations of WS-CM for 3 h with or without NAC. CFSE-labeled K562 cells were then added as target cells and incubated for an additional 5 h. Cells were stained with 7AAD, and flow cytometry was used to gauge the killing of CFSE-labeled K562 cells. Flow cytometry data with percent of killing are shown in the *gated boxes* (**a**). Each point represents the mean ± SD *error bars* of four PBMC donors (**b**). Statistical significance is indicated by *P < 0.05.

Next we measured the levels of cytokines to assess whether NAC reverses the suppression of agonist-mediated select inflammatory cytokine secretion in PBMCs. Cytokine levels of IL-8, TNF, and IL-6 were measured in PBMCs after pretreatment with or without NAC and subsequent co-incubation with WS-CM for 3 h followed by LPS stimulation. At a WS-CM concentration of 1.56 μg/mL, three cytokines (IL-8, TNF, IL-6) were nearly undetectable without NAC treatment (Fig. 3a–c). However, the levels of all three cytokines were markedly increased in response to NAC treatment. After NAC treatment, IL-8 levels increased from 12 to 32 ng/mL and TNF levels increased from 1 to 38 ng/mL (Fig. 3a, b), whereas IL-6 levels increased to 200 ng/mL compared with the control condition (Fig. 3c). These findings show a recovery toward control cytokine levels with the addition of NAC, which was particularly evident with IL-6. Cytolytic function was measured to test the effects of WS-CM on the effector PBMCs to kill K562 cells *in vitro*, with and without NAC. Figure 4a shows the raw data of flow cytometric analysis of K562 cell killing. Cell populations in each dot plot are gated, and the numbers within the gated boxes indicate the percent of K562 cells killed by the effector cells in PBMCs. There is a reduction in K562 cell killing from 18 to 5% at 1.56 μg/mL WS-CM without NAC (Fig. 4a, top panel) and with NAC inclusion percent of K562 cell killing recovered to that of unexposed control condition (Fig. 4a, bottom panel). The combined data show that without NAC pretreatment, K562 cell killing decreased from 14 to 6% with addition of 1.56 μg/mL WS-CM, whereas K562 cell killing was significantly recovered to control levels with NAC treatment in PBMCs exposed to 1.56 μg/mL WS-CM (Fig. 4b).

**Fig. 5:**
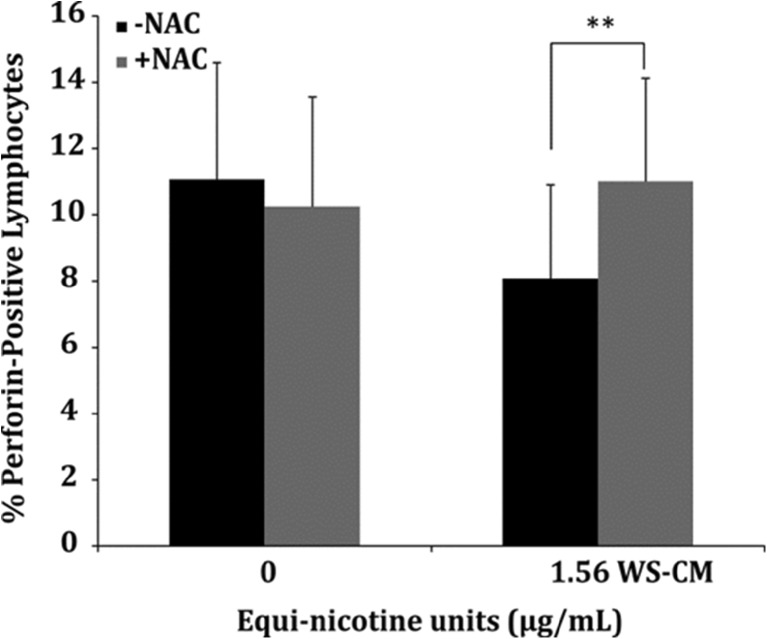
Recovery of perforin-positive lymphocytes with NAC in WS-CM-exposed PBMCs. PBMCs were treated with or without NAC for 1 h followed by co-incubation with WS-CM for 3 h. Perforin levels were determined by cell staining and flow cytometry. The bar graphs are representative data from four different donor PBMCs. Statistical significance is indicated by **P < 0.005.

We measured perforin-positive PBMCs after exposure to WS-CM in the presence and absence of NAC. Perforin is a protease that mediates cytolytic function of (NK) cells and cytotoxic T cells, and is a key enzyme in lysing target cells and thus is critical in regulating immune function. Perforin-positive cells decreased to 30% without NAC treatment compared with NAC treatment in PBMCs exposed to 1.56 μg/mL (Fig. 5). After pretreatment of PBMCs with NAC followed by co-incubation with 1.56 μg/mL WS-CM, perforin-positive cells recovered to the levels of the control condition (Fig. 5).

## DISCUSSION

A previous work has indicated that cigarette smoking induces oxidative stress and inflammation [15, 16]. Several researchers have shown that NAC can reduce inflammation in smokers and that NAC can be potentially used as a therapeutic agent for smoking-related diseases, including cancer and COPD [23, 26, 29, 30]. In this study, we demonstrate that oxidative stress caused by combustible TPPs (WS-CM or TPM) contributes to DNA damage, cell death, suppression of agonist-mediated select inflammatory cytokine secretion, and cytolytic functions of PBMCs in the *ex vivo* model. We showed that reducing oxidative stress by pretreatment with NAC results in improved cell viability, decreased DNA damage, and rescued cytokine secretion by TLR ligands and cytolytic functions in PBMCs.

Several studies have indicated greater levels of oxidative stress in cigarette smokers [31, 32], which is most likely attributed to the high concentration of ROS and nitrogen species (RNS) in cigarette smoke [33–36]. In addition, cigarette smoke has been associated with elevated ROS production by leukocytes in the airways of both acute and chronic smokers [37]. Previous studies have examined the effects of cigarette smoke and NAC on specific immune functions in animal models [24, 25]. NAC is effective in the inhibition of mutagenic properties of ROS [23] as well as modulating cytokine production and inflammation [21, 38]. Here, we measured a dose-dependent increase in ROS using PBMCs exposed to increasing concentrations of WS-CM and TPM, which was restored in the presence of NAC. We also measured a dose-dependent increase in levels of eNOS (nitric oxide synthase) with PBMCs exposed to increasing concentrations of TPM but not with WS-CM (unpublished results).

We have previously reported the dose-dependent increase in cell death and H2AX-positive cells with WS-CM- and TPM-exposed PBMCs [8]. In this study, our findings of increased H2AX-positive cells (increased DNA damage) and increased cell death after exposure to WS-CM added to the evidence that the oxidative stress is triggered by cigarette smoking [39, 40]. Here, we also demonstrated that NAC significantly reduces both DNA damage and cell death in PBMCs exposed to WS-CM.

TLR-stimulated inflammatory responses associated with cytokine expression and secretion were reduced significantly with the combustible TPPs [12–14]. Here we demonstrated that the levels of the cytokines TNF, IL-6, and IL-8 were significantly reduced after exposure to WS-CM and recovered to the control levels in response to NAC treatment.

A functional measure of immune response is cytolysis or target cell killing. Cytolysis is a process which removes infected or cancerous cells with the help of NK cells and cytotoxic T cells. Previously, we and others demonstrated that WS-CM or cigarette smoke-conditioned medium reduces PBMCs and NK cell cytolytic activity [12, 41]. Here we measured target cell killing of K562 cells by effector cells in PBMCs after treatment with WS-CM and showed how NAC significantly increased cytolysis.

Several lines of evidence suggest that NK cells and CD8^+^ cytotoxic T cells mediate cytolysis, which relies on perforin and granzyme-B, mainly by the granule exocytosis pathway in addition to a number of activating and inhibitory receptor-mediated pathways [42, 43]. Decreased cytolysis of NK cells and/or cytotoxic T lymphocytes is associated with reduced perforin expression [12, 41]. Here, we showed that perforin levels are reduced in PBMCs exposed to WS-CM, which was significantly reversed in the presence of an anti-oxidant.

## CONCLUSION

Our data suggest that the oxidative stress caused by combustible TPPs suppressed immune responses in PBMCs as shown by change in levels of cytokines, cytolysis, and increased cytotoxicity. This immune suppression was restored by the addition of the anti-oxidant NAC. Additional work is necessary to define the molecular mechanisms involved in the restoration of oxidative stress and its role in immunomodulating the effects of combustible TPPs.

## Abbreviations

7AAD: 7-Aminoactinomycin D
CBA: Cytometric Bead Array
CFSE: Carboxyfluorescein succinimidyl ester
CSE: Cigarette smoke extract
ELISA: Enzyme-linked immunosorbent assay
IFN: Interferon
IL: Interleukin
LPS: Lipopolysaccharide
NAC: *N*-Acetylcysteine
NK: Natural killer
PBMCs: Peripheral blood mononuclear cells
ROS: Reactive oxygen species
TLR: Toll-like receptor
TPM: Total particulate matter
TPPs: Tobacco product preparations
TNF: Tumor necrosis factor
WS-CM: whole-smoke conditioned medium
